# Tris(2-chloro­benz­yl)[3-(4-methyl­phen­yl)prop-2-enoato-κ*O*]tin(IV)

**DOI:** 10.1107/S1600536811015741

**Published:** 2011-05-07

**Authors:** Thy Chun Keng, Kong Mun Lo, Seik Weng Ng

**Affiliations:** a Department of Chemistry, University of Malaya, 50603 Kuala Lumpur, Malaysia

## Abstract

The Sn^IV^ atom in the title compound, [Sn(C_7_H_6_Cl)_3_(C_10_H_9_O_2_)], exists in a tetra­hedral geometry [Σ C—Sn—C = 341.5 (4)°]. If the doubly bonded carbonyl O atom is taken into account for the coordination sphere of Sn [Sn⋯O = 2.808 (2) Å], the coordination geometry can be described as a *cis*-penta­gonal bipyramid.

## Related literature

Trialkyl­tin(IV) carboxyl­ates contain five-coordinate Sn atoms and are carboxyl­ate-bridged polymers; see: Ng *et al.* (1986 ▶). For the structure of tribenzyl­tin acetate, see: Ferguson *et al.* (1995 ▶).
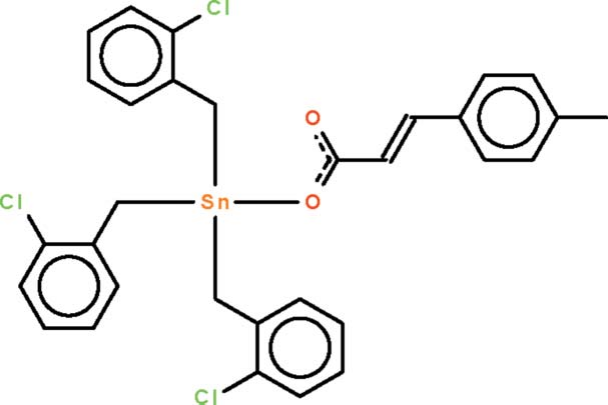


## Experimental

### 

#### Crystal data

[Sn(C_7_H_6_Cl)_3_(C_10_H_9_O_2_)]
*M* *_r_* = 656.57
Triclinic, 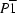

*a* = 10.3162 (1) Å
*b* = 11.0056 (1) Å
*c* = 13.7555 (2) Å
α = 78.7708 (6)°
β = 72.3135 (5)°
γ = 86.5793 (6)°
*V* = 1459.44 (3) Å^3^
*Z* = 2
Mo *K*α radiation
μ = 1.18 mm^−1^
*T* = 100 K
0.45 × 0.35 × 0.25 mm

#### Data collection

Bruker SMART APEX diffractometer
Absorption correction: multi-scan (*SADABS*; Sheldrick, 1996 ▶) *T*_min_ = 0.620, *T*_max_ = 0.758
14046 measured reflections
6689 independent reflections
6056 reflections with *I* > 2σ(*I*)
*R*_int_ = 0.013

#### Refinement

*R*[*F* ^2^ > 2σ(*F* ^2^)] = 0.036
*wR*(*F* ^2^) = 0.106
*S* = 0.98
6689 reflections
335 parameters
H-atom parameters constrained
Δρ_max_ = 1.36 e Å^−3^
Δρ_min_ = −0.82 e Å^−3^

### 

Data collection: *APEX2* (Bruker, 2009 ▶); cell refinement: *SAINT* (Bruker, 2009 ▶); data reduction: *SAINT*; program(s) used to solve structure: *SHELXS97* (Sheldrick, 2008 ▶); program(s) used to refine structure: *SHELXL97* (Sheldrick, 2008 ▶); molecular graphics: *X-SEED* (Barbour, 2001 ▶); software used to prepare material for publication: *publCIF* (Westrip, 2010 ▶).

## Supplementary Material

Crystal structure: contains datablocks global, I. DOI: 10.1107/S1600536811015741/bt5529sup1.cif

Structure factors: contains datablocks I. DOI: 10.1107/S1600536811015741/bt5529Isup2.hkl

Additional supplementary materials:  crystallographic information; 3D view; checkCIF report

## Figures and Tables

**Fig. 1.**
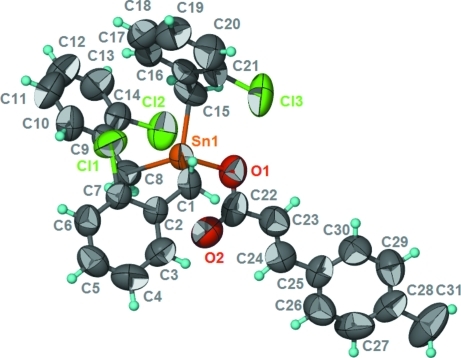
Anisotropic displacement ellipsoid plot (Barbour, 2001) of Sn(C7H6Cl)3(C10H9O2) at the 70% probability level; hydrogen atoms are drawn as spheres of arbitrary radius.

## supplementary crystallographic information

### Comment

Trialkyltin carboxylates generally adopt five-coordinate, carboxylate-bridged structures (Ng *et al.*, 1986), as exemplified by tribenzyltin acetate, which is polymeric with a short and a long Sn–O bond [2.131 (2), 2.559 (2) Å] (Ferguson *et al.*, 1995). In the present 4-cinnamate (Scheme I), the Sn atom adopts a tetrahedral arrangment only. As noted from the sum of C–Sn–C angles at Sn, [Σ C–Sn–C 341.5 (4) °] the geometry is distorted owing to the proximity of the carbonyl O atom [Sn···O 2.808 (2) Å], but a better explanation of the lower coordination status may be attributed to crowding by the three Cl atoms.

### Experimental

Tri(2-chlorobenzyl)tin hydroxide was first prepared by the base hydrolysis of tri(2-chlorobenzyl)tin chloride with 10% sodium hydroxide solution. The hydroxide (0.51 g, 1 mmol) and 4-methylcinnamic acid(0.16 g, 1 mmol) were heated in ethanol (100 ml) until the reactants dissolved completely. The solution was then filtered and a white crystalline solid was obtained upon slow evaporation of the solvent.

### Refinement

Carbon-bound H-atoms were placed in calculated positions (C—H 0.95 to 0.99 Å) and were included in the refinement in the riding model approximation, with *U*(H) set to 1.2–1.5 times *U*_eq_(C).

### Crystal data

**Table:**

[Sn(C_7_H_6_Cl)_3_(C_10_H_9_O_2_)]	*Z* = 2
*M**_r_* = 656.57	*F*(000) = 660
Triclinic, *P*1	*D*_x_ = 1.494 Mg m^−^^3^
Hall symbol: -P 1	Mo *K*α radiation, λ = 0.71073 Å
*a* = 10.3162 (1) Å	Cell parameters from 9997 reflections
*b* = 11.0056 (1) Å	θ = 2.2–28.3°
*c* = 13.7555 (2) Å	µ = 1.18 mm^−^^1^
α = 78.7708 (6)°	*T* = 100 K
β = 72.3135 (5)°	Block, colorless
γ = 86.5793 (6)°	0.45 × 0.35 × 0.25 mm
*V* = 1459.44 (3) Å^3^	

### Data collection

**Table:**

Bruker SMART APEX diffractometer	6689 independent reflections
Radiation source: fine-focus sealed tube	6056 reflections with *I* > 2σ(*I*)
graphite	*R*_int_ = 0.013
ω scans	θ_max_ = 27.5°, θ_min_ = 1.6°
Absorption correction: multi-scan (*SADABS*; Sheldrick, 1996)	*h* = −13→13
*T*_min_ = 0.620, *T*_max_ = 0.758	*k* = −14→14
14046 measured reflections	*l* = −17→17

### Refinement

**Table:**

Refinement on *F*^2^	Primary atom site location: structure-invariant direct methods
Least-squares matrix: full	Secondary atom site location: difference Fourier map
*R*[*F*^2^ > 2σ(*F*^2^)] = 0.036	Hydrogen site location: inferred from neighbouring sites
*wR*(*F*^2^) = 0.106	H-atom parameters constrained
*S* = 0.98	*w* = 1/[σ^2^(*F*_o_^2^) + (0.0634*P*)^2^ + 1.0785*P*] where *P* = (*F*_o_^2^ + 2*F*_c_^2^)/3
6689 reflections	(Δ/σ)_max_ = 0.001
335 parameters	Δρ_max_ = 1.36 e Å^−^^3^
0 restraints	Δρ_min_ = −0.82 e Å^−^^3^

### Fractional atomic coordinates and isotropic or equivalent isotropic displacement parameters (Å^2^)

**Table:**

	*x*	*y*	*z*	*U*_iso_*/*U*_eq_	
Sn1	0.383309 (18)	0.774804 (17)	0.811076 (15)	0.05269 (8)	
Cl2	0.58120 (11)	1.00795 (12)	0.60420 (9)	0.0934 (3)	
Cl1	0.04588 (12)	0.81930 (10)	0.97866 (11)	0.0949 (3)	
Cl3	0.50987 (14)	0.66211 (14)	1.04853 (12)	0.1139 (4)	
O1	0.5625 (2)	0.6896 (3)	0.7465 (2)	0.0774 (7)	
O2	0.4393 (3)	0.6160 (3)	0.6664 (3)	0.0927 (8)	
C1	0.2555 (3)	0.6245 (3)	0.9107 (3)	0.0632 (8)	
H1A	0.3046	0.5455	0.9011	0.076*	
H1B	0.2393	0.6328	0.9838	0.076*	
C2	0.1212 (3)	0.6180 (3)	0.8916 (2)	0.0540 (6)	
C3	0.0931 (4)	0.5256 (3)	0.8458 (3)	0.0753 (9)	
H3	0.1619	0.4670	0.8237	0.090*	
C4	−0.0327 (5)	0.5163 (4)	0.8311 (4)	0.0891 (12)	
H4	−0.0495	0.4513	0.7999	0.107*	
C5	−0.1332 (4)	0.6008 (4)	0.8614 (3)	0.0832 (11)	
H5	−0.2191	0.5952	0.8505	0.100*	
C6	−0.1093 (3)	0.6922 (3)	0.9070 (3)	0.0723 (9)	
H6	−0.1784	0.7506	0.9289	0.087*	
C7	0.0171 (3)	0.6997 (3)	0.9215 (3)	0.0585 (7)	
C8	0.3016 (4)	0.8905 (3)	0.6984 (3)	0.0687 (8)	
H8A	0.3503	0.8726	0.6287	0.082*	
H8B	0.2046	0.8694	0.7140	0.082*	
C9	0.3128 (3)	1.0257 (3)	0.6962 (2)	0.0602 (7)	
C10	0.2005 (4)	1.0943 (4)	0.7405 (4)	0.0845 (11)	
H10	0.1146	1.0548	0.7726	0.101*	
C11	0.2124 (6)	1.2202 (4)	0.7383 (4)	0.1025 (15)	
H11	0.1346	1.2653	0.7695	0.123*	
C12	0.3324 (6)	1.2788 (4)	0.6925 (5)	0.1064 (16)	
H12	0.3382	1.3653	0.6894	0.128*	
C13	0.4454 (5)	1.2142 (4)	0.6505 (4)	0.0890 (12)	
H13	0.5309	1.2546	0.6196	0.107*	
C14	0.4341 (4)	1.0893 (3)	0.6535 (3)	0.0642 (7)	
C15	0.4668 (4)	0.8857 (4)	0.8909 (3)	0.0728 (9)	
H15A	0.5607	0.8575	0.8874	0.087*	
H15B	0.4716	0.9731	0.8544	0.087*	
C16	0.3859 (3)	0.8796 (3)	1.0022 (2)	0.0595 (7)	
C17	0.2901 (4)	0.9715 (3)	1.0334 (3)	0.0714 (9)	
H17	0.2768	1.0391	0.9826	0.086*	
C18	0.2151 (4)	0.9657 (4)	1.1353 (4)	0.0850 (11)	
H18	0.1509	1.0291	1.1540	0.102*	
C19	0.2315 (5)	0.8716 (5)	1.2091 (4)	0.0937 (13)	
H19	0.1797	0.8692	1.2796	0.112*	
C20	0.3234 (5)	0.7785 (4)	1.1826 (3)	0.0888 (12)	
H20	0.3350	0.7117	1.2345	0.107*	
C21	0.3985 (4)	0.7830 (3)	1.0797 (3)	0.0681 (8)	
C22	0.5477 (4)	0.6218 (3)	0.6857 (3)	0.0745 (10)	
C23	0.6762 (4)	0.5491 (4)	0.6446 (3)	0.0814 (10)	
H23	0.7508	0.5526	0.6709	0.098*	
C24	0.6864 (4)	0.4838 (4)	0.5760 (3)	0.0765 (9)	
H24	0.6130	0.4862	0.5474	0.092*	
C25	0.8045 (3)	0.4042 (3)	0.5373 (3)	0.0677 (8)	
C26	0.8045 (4)	0.3506 (5)	0.4566 (4)	0.0935 (13)	
H26	0.7326	0.3678	0.4261	0.112*	
C27	0.9073 (5)	0.2718 (6)	0.4184 (5)	0.1090 (18)	
H27	0.9054	0.2373	0.3607	0.131*	
C28	1.0116 (4)	0.2411 (4)	0.4593 (4)	0.0848 (12)	
C29	1.0152 (4)	0.2971 (5)	0.5390 (3)	0.0881 (12)	
H29	1.0881	0.2799	0.5684	0.106*	
C30	0.9129 (4)	0.3788 (4)	0.5774 (3)	0.0830 (11)	
H30	0.9176	0.4179	0.6320	0.100*	
C31	1.1227 (6)	0.1511 (5)	0.4187 (6)	0.144 (3)	
H31A	1.0819	0.0788	0.4077	0.216*	
H31B	1.1729	0.1246	0.4694	0.216*	
H31C	1.1853	0.1919	0.3528	0.216*	

### Atomic displacement parameters (Å^2^)

**Table:**

	*U*^11^	*U*^22^	*U*^33^	*U*^12^	*U*^13^	*U*^23^
Sn1	0.04486 (12)	0.05104 (12)	0.05715 (13)	−0.00428 (8)	−0.01183 (8)	−0.00238 (8)
Cl2	0.0703 (6)	0.1004 (7)	0.0886 (6)	0.0104 (5)	−0.0013 (5)	−0.0074 (5)
Cl1	0.0817 (6)	0.0811 (6)	0.1307 (9)	0.0014 (5)	−0.0253 (6)	−0.0513 (6)
Cl3	0.1010 (8)	0.1149 (9)	0.1186 (9)	0.0513 (7)	−0.0362 (7)	−0.0146 (7)
O1	0.0512 (12)	0.0772 (16)	0.0937 (18)	0.0023 (11)	−0.0073 (12)	−0.0152 (14)
O2	0.095 (2)	0.0801 (18)	0.107 (2)	0.0082 (15)	−0.0302 (17)	−0.0286 (16)
C1	0.0472 (14)	0.0567 (16)	0.0752 (19)	−0.0017 (12)	−0.0153 (14)	0.0082 (14)
C2	0.0491 (14)	0.0467 (13)	0.0571 (15)	−0.0045 (11)	−0.0084 (12)	0.0011 (11)
C3	0.073 (2)	0.0644 (19)	0.083 (2)	−0.0018 (16)	−0.0114 (18)	−0.0208 (17)
C4	0.097 (3)	0.089 (3)	0.088 (3)	−0.024 (2)	−0.026 (2)	−0.026 (2)
C5	0.068 (2)	0.093 (3)	0.090 (3)	−0.020 (2)	−0.031 (2)	−0.002 (2)
C6	0.0517 (16)	0.0675 (19)	0.091 (2)	−0.0009 (14)	−0.0166 (16)	−0.0058 (17)
C7	0.0512 (15)	0.0496 (14)	0.0696 (18)	−0.0047 (11)	−0.0118 (13)	−0.0075 (13)
C8	0.082 (2)	0.0557 (16)	0.077 (2)	−0.0067 (15)	−0.0395 (18)	−0.0059 (15)
C9	0.0667 (18)	0.0540 (15)	0.0601 (16)	−0.0020 (13)	−0.0249 (14)	−0.0006 (13)
C10	0.068 (2)	0.078 (2)	0.097 (3)	0.0055 (18)	−0.019 (2)	−0.003 (2)
C11	0.108 (4)	0.073 (3)	0.116 (4)	0.025 (3)	−0.020 (3)	−0.022 (2)
C12	0.130 (4)	0.056 (2)	0.123 (4)	0.006 (2)	−0.027 (3)	−0.013 (2)
C13	0.099 (3)	0.063 (2)	0.095 (3)	−0.015 (2)	−0.020 (2)	−0.0021 (19)
C14	0.0670 (18)	0.0592 (17)	0.0602 (17)	0.0011 (14)	−0.0155 (14)	−0.0023 (13)
C15	0.071 (2)	0.085 (2)	0.0625 (18)	−0.0279 (18)	−0.0212 (16)	−0.0016 (16)
C16	0.0558 (16)	0.0635 (17)	0.0624 (17)	−0.0124 (13)	−0.0260 (13)	−0.0024 (13)
C17	0.074 (2)	0.0535 (16)	0.093 (3)	−0.0059 (15)	−0.0395 (19)	−0.0059 (16)
C18	0.073 (2)	0.076 (2)	0.104 (3)	−0.0016 (18)	−0.016 (2)	−0.030 (2)
C19	0.092 (3)	0.101 (3)	0.077 (2)	−0.005 (2)	−0.004 (2)	−0.023 (2)
C20	0.102 (3)	0.092 (3)	0.062 (2)	0.003 (2)	−0.021 (2)	0.0042 (19)
C21	0.0612 (18)	0.073 (2)	0.0683 (19)	0.0097 (15)	−0.0239 (15)	−0.0036 (15)
C22	0.0569 (18)	0.0645 (19)	0.082 (2)	0.0040 (15)	−0.0003 (17)	−0.0009 (17)
C23	0.071 (2)	0.088 (3)	0.086 (3)	−0.0059 (19)	−0.0226 (19)	−0.016 (2)
C24	0.075 (2)	0.074 (2)	0.073 (2)	−0.0107 (17)	−0.0158 (18)	−0.0010 (17)
C25	0.0546 (17)	0.0608 (17)	0.0699 (19)	−0.0012 (13)	0.0012 (14)	−0.0012 (15)
C26	0.065 (2)	0.124 (4)	0.097 (3)	0.000 (2)	−0.024 (2)	−0.034 (3)
C27	0.077 (3)	0.137 (4)	0.124 (4)	−0.012 (3)	−0.011 (3)	−0.077 (4)
C28	0.064 (2)	0.067 (2)	0.104 (3)	−0.0097 (16)	0.011 (2)	−0.026 (2)
C29	0.062 (2)	0.108 (3)	0.083 (3)	0.008 (2)	−0.0141 (19)	−0.007 (2)
C30	0.081 (2)	0.101 (3)	0.065 (2)	0.003 (2)	−0.0105 (18)	−0.030 (2)
C31	0.088 (3)	0.096 (4)	0.204 (7)	0.008 (3)	0.035 (4)	−0.054 (4)

### Geometric parameters (Å, °)

**Table:**

Sn1—O1	2.050 (2)	C13—H13	0.9500
Sn1—C8	2.152 (3)	C15—C16	1.496 (5)
Sn1—C1	2.152 (3)	C15—H15A	0.9900
Sn1—C15	2.158 (3)	C15—H15B	0.9900
Cl2—C14	1.735 (4)	C16—C21	1.382 (4)
Cl1—C7	1.741 (3)	C16—C17	1.405 (5)
Cl3—C21	1.736 (4)	C17—C18	1.372 (6)
O1—C22	1.270 (5)	C17—H17	0.9500
O2—C22	1.234 (5)	C18—C19	1.344 (7)
C1—C2	1.494 (4)	C18—H18	0.9500
C1—H1A	0.9900	C19—C20	1.379 (6)
C1—H1B	0.9900	C19—H19	0.9500
C2—C7	1.375 (4)	C20—C21	1.385 (5)
C2—C3	1.383 (5)	C20—H20	0.9500
C3—C4	1.385 (6)	C22—C23	1.520 (5)
C3—H3	0.9500	C23—C24	1.270 (6)
C4—C5	1.375 (7)	C23—H23	0.9500
C4—H4	0.9500	C24—C25	1.482 (5)
C5—C6	1.355 (6)	C24—H24	0.9500
C5—H5	0.9500	C25—C26	1.355 (6)
C6—C7	1.386 (5)	C25—C30	1.382 (6)
C6—H6	0.9500	C26—C27	1.373 (7)
C8—C9	1.493 (4)	C26—H26	0.9500
C8—H8A	0.9900	C27—C28	1.356 (7)
C8—H8B	0.9900	C27—H27	0.9500
C9—C14	1.378 (5)	C28—C29	1.369 (7)
C9—C10	1.388 (5)	C28—C31	1.515 (6)
C10—C11	1.392 (6)	C29—C30	1.391 (6)
C10—H10	0.9500	C29—H29	0.9500
C11—C12	1.345 (8)	C30—H30	0.9500
C11—H11	0.9500	C31—H31A	0.9800
C12—C13	1.360 (7)	C31—H31B	0.9800
C12—H12	0.9500	C31—H31C	0.9800
C13—C14	1.378 (5)		
			
O1—Sn1—C8	113.63 (14)	C16—C15—H15A	108.9
O1—Sn1—C1	103.75 (11)	Sn1—C15—H15A	108.9
C8—Sn1—C1	115.95 (13)	C16—C15—H15B	108.9
O1—Sn1—C15	96.09 (14)	Sn1—C15—H15B	108.9
C8—Sn1—C15	110.81 (14)	H15A—C15—H15B	107.7
C1—Sn1—C15	114.71 (14)	C21—C16—C17	116.3 (3)
C22—O1—Sn1	111.2 (2)	C21—C16—C15	122.3 (3)
C2—C1—Sn1	114.3 (2)	C17—C16—C15	121.4 (3)
C2—C1—H1A	108.7	C18—C17—C16	121.5 (4)
Sn1—C1—H1A	108.7	C18—C17—H17	119.3
C2—C1—H1B	108.7	C16—C17—H17	119.3
Sn1—C1—H1B	108.7	C19—C18—C17	120.8 (4)
H1A—C1—H1B	107.6	C19—C18—H18	119.6
C7—C2—C3	116.0 (3)	C17—C18—H18	119.6
C7—C2—C1	122.6 (3)	C18—C19—C20	120.0 (4)
C3—C2—C1	121.3 (3)	C18—C19—H19	120.0
C2—C3—C4	121.8 (4)	C20—C19—H19	120.0
C2—C3—H3	119.1	C19—C20—C21	119.5 (4)
C4—C3—H3	119.1	C19—C20—H20	120.2
C5—C4—C3	120.1 (4)	C21—C20—H20	120.2
C5—C4—H4	120.0	C16—C21—C20	121.9 (4)
C3—C4—H4	120.0	C16—C21—Cl3	119.8 (3)
C6—C5—C4	119.7 (4)	C20—C21—Cl3	118.3 (3)
C6—C5—H5	120.2	O2—C22—O1	122.3 (3)
C4—C5—H5	120.2	O2—C22—C23	125.8 (4)
C5—C6—C7	119.4 (4)	O1—C22—C23	111.9 (4)
C5—C6—H6	120.3	C24—C23—C22	121.7 (4)
C7—C6—H6	120.3	C24—C23—H23	119.1
C2—C7—C6	123.1 (3)	C22—C23—H23	119.1
C2—C7—Cl1	118.5 (2)	C23—C24—C25	124.7 (4)
C6—C7—Cl1	118.4 (3)	C23—C24—H24	117.6
C9—C8—Sn1	113.4 (2)	C25—C24—H24	117.6
C9—C8—H8A	108.9	C26—C25—C30	117.6 (4)
Sn1—C8—H8A	108.9	C26—C25—C24	116.8 (4)
C9—C8—H8B	108.9	C30—C25—C24	125.6 (4)
Sn1—C8—H8B	108.9	C25—C26—C27	120.6 (4)
H8A—C8—H8B	107.7	C25—C26—H26	119.7
C14—C9—C10	115.9 (3)	C27—C26—H26	119.7
C14—C9—C8	122.7 (3)	C28—C27—C26	122.9 (4)
C10—C9—C8	121.3 (3)	C28—C27—H27	118.5
C11—C10—C9	120.8 (4)	C26—C27—H27	118.5
C11—C10—H10	119.6	C27—C28—C29	117.1 (4)
C9—C10—H10	119.6	C27—C28—C31	122.6 (5)
C12—C11—C10	121.0 (4)	C29—C28—C31	120.2 (5)
C12—C11—H11	119.5	C28—C29—C30	120.5 (4)
C10—C11—H11	119.5	C28—C29—H29	119.7
C11—C12—C13	120.0 (4)	C30—C29—H29	119.7
C11—C12—H12	120.0	C29—C30—C25	121.1 (4)
C13—C12—H12	120.0	C29—C30—H30	119.5
C14—C13—C12	119.1 (4)	C25—C30—H30	119.5
C14—C13—H13	120.5	C28—C31—H31A	109.5
C12—C13—H13	120.5	C28—C31—H31B	109.5
C9—C14—C13	123.2 (4)	H31A—C31—H31B	109.5
C9—C14—Cl2	118.7 (3)	C28—C31—H31C	109.5
C13—C14—Cl2	118.1 (3)	H31A—C31—H31C	109.5
C16—C15—Sn1	113.5 (2)	H31B—C31—H31C	109.5
			
C8—Sn1—O1—C22	−59.1 (3)	C12—C13—C14—Cl2	−177.4 (4)
C1—Sn1—O1—C22	67.7 (3)	O1—Sn1—C15—C16	−131.5 (3)
C15—Sn1—O1—C22	−175.0 (3)	C8—Sn1—C15—C16	110.3 (3)
O1—Sn1—C1—C2	−126.3 (2)	C1—Sn1—C15—C16	−23.3 (3)
C8—Sn1—C1—C2	−1.0 (3)	Sn1—C15—C16—C21	81.2 (4)
C15—Sn1—C1—C2	130.2 (3)	Sn1—C15—C16—C17	−97.3 (3)
Sn1—C1—C2—C7	−73.8 (3)	C21—C16—C17—C18	1.0 (5)
Sn1—C1—C2—C3	108.5 (3)	C15—C16—C17—C18	179.6 (3)
C7—C2—C3—C4	−0.1 (5)	C16—C17—C18—C19	0.2 (6)
C1—C2—C3—C4	177.7 (4)	C17—C18—C19—C20	−0.7 (7)
C2—C3—C4—C5	0.6 (7)	C18—C19—C20—C21	0.1 (8)
C3—C4—C5—C6	−0.9 (7)	C17—C16—C21—C20	−1.6 (5)
C4—C5—C6—C7	0.6 (6)	C15—C16—C21—C20	179.8 (4)
C3—C2—C7—C6	−0.2 (5)	C17—C16—C21—Cl3	177.8 (3)
C1—C2—C7—C6	−178.0 (3)	C15—C16—C21—Cl3	−0.9 (5)
C3—C2—C7—Cl1	−179.4 (3)	C19—C20—C21—C16	1.1 (7)
C1—C2—C7—Cl1	2.8 (4)	C19—C20—C21—Cl3	−178.3 (4)
C5—C6—C7—C2	−0.1 (6)	Sn1—O1—C22—O2	3.4 (5)
C5—C6—C7—Cl1	179.2 (3)	Sn1—O1—C22—C23	−174.9 (2)
O1—Sn1—C8—C9	−108.4 (3)	O2—C22—C23—C24	7.7 (7)
C1—Sn1—C8—C9	131.5 (3)	O1—C22—C23—C24	−174.0 (4)
C15—Sn1—C8—C9	−1.5 (3)	C22—C23—C24—C25	−175.8 (3)
Sn1—C8—C9—C14	74.2 (4)	C23—C24—C25—C26	−173.6 (4)
Sn1—C8—C9—C10	−103.9 (4)	C23—C24—C25—C30	7.5 (6)
C14—C9—C10—C11	1.5 (6)	C30—C25—C26—C27	1.5 (7)
C8—C9—C10—C11	179.8 (4)	C24—C25—C26—C27	−177.5 (4)
C9—C10—C11—C12	0.6 (8)	C25—C26—C27—C28	1.4 (8)
C10—C11—C12—C13	−2.2 (9)	C26—C27—C28—C29	−3.1 (8)
C11—C12—C13—C14	1.6 (8)	C26—C27—C28—C31	178.2 (5)
C10—C9—C14—C13	−2.2 (5)	C27—C28—C29—C30	1.9 (7)
C8—C9—C14—C13	179.6 (4)	C31—C28—C29—C30	−179.4 (4)
C10—C9—C14—Cl2	175.9 (3)	C28—C29—C30—C25	0.9 (7)
C8—C9—C14—Cl2	−2.4 (4)	C26—C25—C30—C29	−2.7 (6)
C12—C13—C14—C9	0.7 (7)	C24—C25—C30—C29	176.3 (4)
